# Death Associated Protein Kinase 1 (DAPK1): A Regulator of Apoptosis and Autophagy

**DOI:** 10.3389/fnmol.2016.00046

**Published:** 2016-06-23

**Authors:** Pratibha Singh, Palaniyandi Ravanan, Priti Talwar

**Affiliations:** Apoptosis and Cell Survival Research Laboratory, Department of Bio-Sciences, School of Bio Sciences and Technology, Vellore Institute of Technology (VIT) UniversityVellore, Tamil Nadu, India

**Keywords:** death-associated protein kinase 1, autophagy, apoptosis, cancer, neurodegenerative disease

## Abstract

Death-Associated Protein Kinase 1 (DAPK1) belongs to a family of five serine/threonine (Ser/Thr) kinases that possess tumor suppressive function and also mediate a wide range of cellular processes, including apoptosis and autophagy. The loss and gain-of–function of DAPK1 is associated with various cancer and neurodegenerative diseases respectively. In recent years, mechanistic studies have broadened our knowledge of the molecular mechanisms involved in DAPK1-mediated autophagy/apoptosis. In the present review, we have discussed the structural information and various cellular functions of DAPK1 in a comprehensive manner.

## Introducing DAPK1

Death-associated protein kinase 1 (DAPK1), a part of a family of Ser/Thr kinase, was originally isolated in an unbiased antisense based genetic screen for genes whose protein products were imperative for interferon Gamma (IFN-γ) induced death in HeLa cells (Deiss et al., 1995), and identified by a functional cloning based on its involvement in interferon-γ-induced apoptosis (Bialik and Kimchi, 2006).

DAPK1 is an important regulator of cell death and autophagy which act as a critical component in the ER stress-induced cell death pathway (Gade et al., 2014). It is a stress-responsive serine/threonine (Ser/Thr) kinase (Hainsworth et al., 2010), which constitutes a critical integration point in ER stress signaling, transmitting these signals into two distinct directions, caspase activation and autophagy, leading to cell death (Gozuacik et al., 2008). DAPK1 is a mediator of pro-apoptotic pathway, involved in multiple cell death processes induced by various internal and external apoptotic stimulants (Celik et al., 2015). This pro-apoptotic Ser/Thr kinase regulates both type I apoptotic (caspase-dependent) and type II autophagic (caspase-independent) cell death signal (Shohat et al., 2001). On the other hand, DAPK1 is a tumor suppressor gene, known to suppress tumor growth and metastasis by promoting autophagy and apoptosis (Bialik and Kimchi, 2006).

It has been hypothesized that DAPK1 play a role in perinatal brain injury via the observation of highly expressed DAPK1 mRNA level in the developing brain (Nair et al., 2013). In a recent study, DAPK1 is identified as a new component of the neuronal death signaling complex (NDC; Lai et al., 2011), which act as a signaling amplifier of N-methyl-D-aspartate (NMDA) receptors at extrasynaptic sites for mediating brain damage in stroke (Tu et al., 2010). Cerebral ischemia recruits DAPK1 into the NMDA receptor complex through its direct binding to the amino acid residues 1292–1304 of the N-methyl D-aspartate receptor subtype 2B (NR2B) carboxyl terminus (NR2B-CT_1292–1304_). This direct DAPK1-NR2B interaction thereby potentiating NR2BR activity via triggering the DAPK1-mediated phosphorylation of NR2B subunit at Ser-1303 (Lai et al., 2011) inducing injurious Ca^2+^ influx through NMDA receptor channels, results in an irreversible neuronal death. Here DAPK1 seems to be a promising target for drug development, especially with the aim of preventing the neuronal apoptosis after stroke as it has a central role in neuronal cell death (Bialik and Kimchi, 2004) and represents a relatively unique enzyme in the protein kinase superfamily whose biological functions are related to both signal-mediated apoptosis and autophagy (Stevens and Hupp, 2008).

The DAPK1 gene is quite conserved in evolution from various invertebrates, such as *C. elegans* (Tong et al., 2009), to chordates and mammals, maps to mouse chromosome 13 and human chromosome 9 and transcribed into a single mRNA of 6.3 kb encoded for a structurally unique 160-kD Ca^2+^/calmodulin (CaM)-dependent serine-threonine kinase (Deiss et al., 1995; Nair et al., 2013). DAPK1 is the largest one in the DAPK protein family, consisting of 1430 amino acids (Refseq: accession NM_004938.2), contains Ca^2+^/CaM autoregulatory domain, 10 ankyrin repeats, 2 putative P-loop consensus sites, Ras of complex proteins, C-terminal of ROC (ROC-COR) domains, which overlap with a cytoskeletal-binding region, a death domain and a serine-rich C-terminal tail (Shiloh et al., 2014); whose phosphorylation activity is known to be responsible for certain forms of apoptotic cell death (Deiss et al., 1995; Cohen et al., 1997, 1999; Kissil et al., 1997).

## DAPK1 Structure and Function

Drug design is mainly being actively carried out within the DAPK family on the basis of active conformational structure of the DAPK1 (Tereshko et al., 2001; Velentza et al., 2001, 2002, 2003; Bialik and Kimchi, 2004; Yamakawa et al., 2004; McNamara et al., 2009; Okamoto et al., 2009, 2010). More than 50 crystal structures of DAPK1, reported in the Protein Data Bank (PDB), are crystallized with their respective inhibitors with a resolution higher than 2.5 Angstroms.

**Figure 1:**
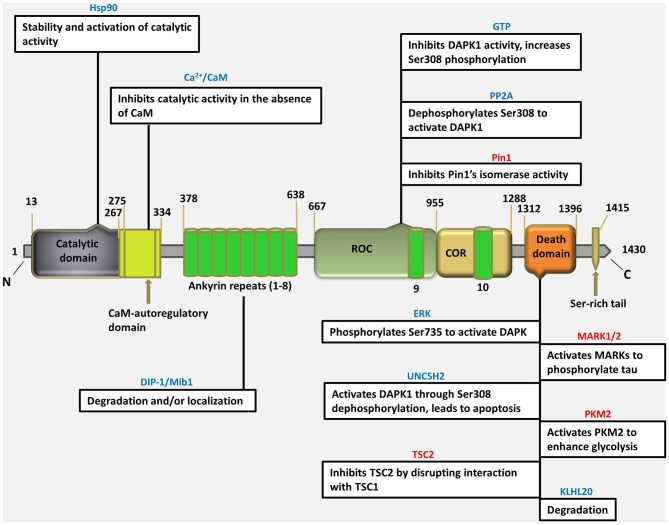
**Schematic depiction of multi-domain organization of death associated protein kinase 1 (DAPK1) protein with amino acid numbering (http://www.uniprot.org/uniprot/P53355), showing different types of interacting proteins/molecules of the various DAPK1 domains and their known or proposed functions.** (Blue colored text indicates an interaction that regulate DAPK1, pink colored text indicates an effector of DAPK1).

The DAPK1 crystal structure, which can be used as the starting point for designing of bioavailable protein kinase inhibitor, is an excellent example of a small-molecule fragment bound to the kinase domain allowing *in vivo* and *in vitro* target validation studies to be performed. However, although initial target validation evidence supports DAPK1 as a drug discovery target for neurological disorders, no clinically promising small-molecule DAPK1 inhibitors have yet been discovered. Therefore, the attractive treatment option for perinatal brain injury is the development of a domain specific small molecule inhibitors for DAPK1 with reduced adverse effects, can easily be administered and screened for specificity and capacity of binding with a target. It is important to gain insight into to understand how the DAPK1, a multi-domain protein operates in a cellular context, and how its dysfunction leads to disease (Nair et al., 2013). For this purpose, the structural studies have been performed describing the precise spatial arrangement of DAPK1 domains and their known or proposed functions (Figure 1).

### The Catalytic Domain: Structure and Regulation

**Figure 2:**
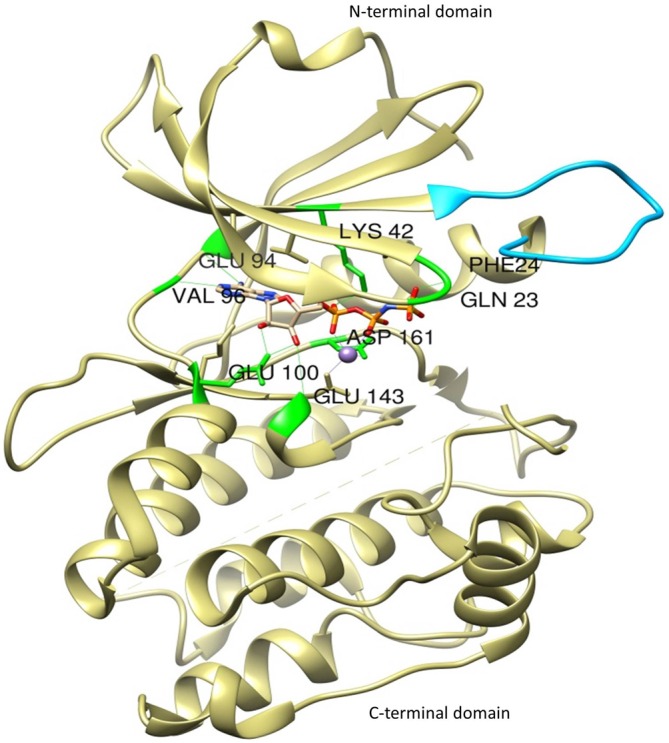
**The crystal structure of the catalytic domain of DAPK1 with ligand Phosphoaminophosphonic Acid-Adenylate Ester (ANP) and Manganese (II) ion showing basic loop (blue) and H-bond interactions with conserved amino acid residues (green)**.

The most thoroughly characterized catalytic domain structure of DAPK1 (PDB ID: 1IG1) was determined at 1.5 Å resolution and has provided structural hints as to DAPK1’s mechanism of activation, interactions with substrates, and potential inhibitor design (Tereshko et al., 2001; Bialik and Kimchi, 2004; Figure 2). According to this structure, DAPK1 exhibits the canonical kinase fold, consisting of a small β-stranded N-terminal domain, and a larger helix-rich C-terminal domain, connected by a hinge region. The DAPK1 contains catalytic domain at its N-terminus composed of the typical 11 subunits. In addition, in many of the available PDB structures, the catalytic domain was crystallized as homodimer and this interesting dimerization profile was confirmed by non-covalent nano-electrospray ionization mass spectrometry (Zimmermann et al., 2010). Another outstanding feature of DAPK1 structure is positioned on the upper lobe of the catalytic domain, which is a highly ordered short segment of mostly positively charged residues, termed as “basic loop” (residues 45–56; Tereshko et al., 2001). These 12 amino acids are a conserved feature of the DAPK family members thus referred to as “the fingerprint” of the family (Inbal et al., 2000). Of note, the basic residues mutation in the loop did not affect the *K_m_* of a peptide substrate (Velentza et al., 2001), indicates indirect involvement in substrate binding rather may be involved in other functions. However, this loop was shown to mediate not only DAPK1 homodimerization as well as homooligomerization via their shared basic loop motif, but also facilitate physical and functional interactions. Here the crystal structure of catalytic domain shows that the kinase is constitutively in the “closed”, active conformation so this feature states that there is no evidence that DAPK1 require phosphorylation of an activation loop, a mechanism common to many other kinases (Nolen et al., 2004). In fact, no activating phosphorylation events on the catalytic domain of DAPK1 have been reported so far.

Interestingly, DAPK1 lack His-Arg-Asp (HRD) motif that require activation loop phosphorylation, common to other kinases in which the conserved arginine in this motif creates a salt bridge with the phosphorylated residue in the activation loop. In DAPK1, a change at residue 138 where the arginine is replaced by a conserved phenylalanine, substitutes phosphorylation dependent active-site interactions with a local hydrophobic core that maintains an active kinase conformation. According to active site analysis of the X-ray crystallographic structure of human DAPK1 complex with respective inhibitor (PDB ID: 1IG1) using Chimera 1.11 version, the active sites of DAPK1 accommodates certain highly conserved amino acid residues such as Gln23, Phe24, Lys42, Glu94, Val96, Glu100, Glu146 and Asp161; involved in H-bond interaction with their respective ligands and hydrophobic interactions includes Leu19, Val27, Ile77, Met146 and Ile160. Here, the hydrogen bond interactions with Val96 and Glu94 are especially important since they reside in a hydrophobic enclosure. There are some additional hydrophobic interactions with Ile77 and Leu93. The amino terminal lobe of the kinase domain contains an interaction surface for the chaperon heat shock protein 90 (HSP90), which recognizes specific neutral and positively charged residues within the alphaC-beta4 loop. This interaction with Hsp90 facilitates kinase maturation, activity and stability. Inhibition of HSP90 leads to degradation of DAPK1 (Nair et al., 2013).

The kinase domain of DAPK1 is followed by a Ca^2+^/CaM autoregulatory domain, which suppresses catalytic activity by binding to the catalytic cleft, and functions as a pseudosubstrate (Shani et al., 2001; Shohat et al., 2001). The autoregulatory domain binds to the catalytic cleft in the absence of CaM and blocks access of exogenous substrates, thus inhibits DAPK1 function. The binding of CaM to the autoregulatory domain pulls it away from catalytic cleft and enables substrate phosphorylation. Thus, due to this inhibitory role, a mutant DAPK1 lacking this domain (ΔCaM mutant) is constitutively active in both *in vitro* and *in vivo* (Inbal et al., 2000). There are several phosphorylation sites, located within the CaM autoregulated domain, two of which Ser289 and Ser308, regulate the DAPK1 activity. Here dephosphorylation of Ser308 increases the affinity for the CaM thereby promoting the catalytic activity at low CaM levels (Nair et al., 2013). Hence, DAPK1 is regulated by a double-locking mechanism as full activation of DAPK1 requires two events: first, binding of Ca^2+^-activated CaM to the autoregulatory/CaM-binding segment (Cohen et al., 1997; Inbal et al., 2000; Lai et al., 2005); second, dephosphorylation of Ser308 (Shani et al., 2001; Shohat et al., 2001). It has been shown that the deletion of CaM-binding domain or substitution of Ser308 to Ala, generates constitutively active form of DAPK1 thereby exhibiting greater Ca^2+^ independent catalytic activity *in vitro* and strong killing potential *in vivo* (Cohen et al., 1997; Inbal et al., 2000; Shani et al., 2001; Shohat et al., 2001).

In summary, activation of DAPK1 requires two events: one, common to all CaM dependent kinases, is binding of Ca^2+^-activated CaM to the autoregulatory CaM-binding segment, which pulls this domain out from the catalytic cleft. The other, unique to DAPK1, is dephosphorylation of Ser308, increases the affinity to CaM, facilitates the removal of the autoregulatory domain from the catalytic cleft and promotes catalytic activity at low Ca^2+^ level.

### The Extracatalytic Domains

#### Ankyrin Repeats

DAPK1, at 160kDa, contains a large C- terminal extension with multiple functional extensions with multiple functional domains. It contains a series of 10 ankyrin repeats, which facilitate protein-protein communications and are implicated mostly in DAPK1 degradation (Nair et al., 2013). An alternatively spliced product, s-DAPK-1, lacking the DAPK1 kinase domain regulates the steady-state levels of the full-length DAPK1 and is implicated in proteasome-independent degradation pathway for DAPK1(Lin et al., 2009). The ankyrin repeats also facilitate the degradation of DAPK1 via the ubiquitin-proteasome pathway. The mindbomb E3 ubiquitin ligase 1 (MIB-1), also known as DAPK-1 interacting protein i.e., DIP-1, is able to actively ubiquitinate and degrade DAPK1 via interaction with ankyrin repeats, affects its stability by mediating its proteasomal-dependent degradation, or alternatively, helps in subcellular localization (Jin et al., 2002). As DAPK1 interacts with HSP90 through its kinase domain, the E3 ubiquitin ligase carboxyl terminus of HSC70-interacting protein (CHIP), which facilitates the ubiquitination of HSP90-interacting proteins, will also induce DAPK1 degradation (Connell et al., 2001; Zhang et al., 2007). Deletion of the ankyrin repeats, which is protein-protein interaction domain prominent in cytoskeletal proteins, can cause mislocalization of DAPK1 from actin filaments to focal adhesions.

#### ROC-COR Domain

Recently, DAPK1 was shown to be related to the ROCO family of proteins, as it shares weak yet significant homology with the ROCO proteins (Carlessi et al., 2011), which are multidomain proteins, mostly kinases, characterized by having two domain that always appear in tandem: the Ras of complex protein (ROC) domain- a guanosine triphosphate (GTP)ase domain similar to Ras and other small G-proteins- and immediately downstream, the C-terminal of ROC (COR) domain. These domains are located between 667–1288 residues of DAPK1. DAPK1 binds GTP through a P-loop motif present in the ROC domain (between the residues 695–702). The ROC-COR domains promote GTP hydrolysis which indicates that DAPK1 is a GTP-binding protein with intrinsic GTPase activity. GTP-binding is believed to suppress DAPK1 activity by enhancing the inhibitory phospho-Ser308 within the same molecule, through intramolecular signaling. Here GTP is hydrolyzed to GDP by the ROC domain, resulting in some conformational changes that are propagated to the N-terminus, which ultimately decreases Ser308 autophosphorylation. Therefore this domain provides an additional mechanism for the regulation of DAPK1 activity, in a unique manner (Shiloh et al., 2014). As in other ROCO family proteins, the ROC domain was also shown to mediate DAPK1 homodimerization by both its kinase domain and the ROC domain (Carlessi et al., 2011).

The ROC-COR domains, also called as cytoskeletal localization domain, and can mediate an interaction with the phosphor-Ser/Thr-directed peptidyl prolyl isomerase 1 (Pin1), which is a critical signaling protein that modulates the function, stability and/or localization of numerous phosphoproteins through isomerization of proline that follows a phosphorylation site (Lee et al., 2011). DAPK1 phosphorylates and inactivates Pin1, thereby suppressing its cellular functions, including its ability to induce cellular transformation (Shiloh et al., 2014). Thus the ROC-COR domain serves not only to regulate DAPK1 catalytic activity, but can also mediate cellular functions through protein-protein interactions.

#### Death Domain

The death domain is a protein-protein interaction mediating domain that is common to many apoptosis-promoting proteins, such as Fas, TNF receptor type 1-associated death domain protein (TRADD), Fas-associated protein with death domain (FADD) and Tumor necrosis factor (TNF) receptor. In DAPK1, death domain is largely associated with protein-protein interactions, kinase activity and apoptotic functions. DAPK1’s death domain is located near its C-terminus between residues 1312–1396, followed by a 17-aa tail rich in Serine residues, a common feature in other death domain-containing proteins (Feinstein et al., 1995). The death domain of DAPK1 was suggested to mediate its role in TNF-α and Fas-induced apoptosis, as transfection of death domain fragment reduced TNF-α and Fas-induced apoptosis, only in cells that expressed endogenous DAPK1. Thus the deletion of the death domain affects the apoptotic functions of TNF-α and Fas-induced cell death (Cohen et al., 1999).

The death domain was shown to mediate an interaction between extracellular-signal-regulated (ERK) and DAPK1 (Chen et al., 2005). ERK causes an increase in DAPK1 catalytic activity via phosphorylating DAPK1 on Ser735. In the mutant DAPK1 lacking the death domain, the activation of DAPK1 by ERK was eliminated, highlighting a mechanism through which the death domain may be necessary for full DAPK1 activity in cells (Shiloh et al., 2014).

DAPK1’s death domain was also shown to mediate interactions that promote degradation of DAPK1 by the proteasome. It binds a beta -turn-beta (BTB)/Kelch protein, Kelch-like protein 20 ( KLHL20) that act as an adaptor for the Cullin3-ROC1 ubiquitin E3 ligase complex. IFN results in the sequestration of KLHL20 away from DAPK1 cause high protein level of DAPK1 upon IFN treatment (Lee et al., 2010).

The death domain was also shown to mediate an interaction between DAPK1 and microtubule (MT) affinity regulating kinases (MARK1/2). MARK is a Ser/Thr kinase that destabilizes microtubules by phosphorylating tau (Wu et al., 2011). DAPK1 helps in relieving an inhibitory intramolecular interaction between the catalytic and spacer regions of MARK1/2 by activating it through the binding to its spacer region. Thus DAPK1 promotes MARK1/2-induced MT destabilization and neuronal differentiation which may also cause tau toxicity and neurodegeneration.

A second effector, pyruvate kinase isoform M2 (PKM2) is an isoform of the glycolytic enzyme, binds the death domain, which is commonly expressed in cancer. DAPK1 leads to an enhanced glycolytic rate and lactate production by activating PKM2’s activity *in vitro* and *in vivo* (Mor et al., 2012). Thus a particularly interesting aspect of the interaction of DAPK1 with both MARK1/2 and PKM2 is that activating effect of DAPK1 on these proteins is mediated solely through binding, and is not dependent on DAPK1 catalytic activity (Wu et al., 2011; Mor et al., 2012).

Another interaction is between DAPK1’s death-domain and death-domain containing protein Unc-5 homolog 2 (UNC5H2), a dependence receptor which triggers apoptosis in the absence of its ligand netrin-1. This interaction is probably mediated through domains of both proteins as well as through additional domains and was shown to be necessary for UNC5H5-induced apoptosis, as co-transfection of a dominant-negative DAPK1 mutant together with UNC5H2 decreased cell death (Llambi et al., 2005). Remarkably, overexpression of UNC5H2 activates DAPK1 via dephosphorylation of Ser308 in a netrin-1 dependent manner. Thus, this is another example of a role for the death domain in promoting activation of DAPK1.

An additional interaction of DAPK1’s death domain is with tuberous sclerosis 2 (TSC2), a negative regulator of the mechanistic target of energy, growth factors and nutrient that controls biosynthetic pathways and multiple cell growth (Stevens et al., 2009). TSC2, as part of the TSC1/TSC2 dimer, act as a GAP for the small GTPase protein Ras homolog enriched in brain (Rheb) and activates mechanistic target of rapamycin (mTOR) in its GTP–bound state. DAPK1’s interaction with TSC2 interferes with TSC1 binding, and hence its ability to suppress mTOR. Thus, there is a positive association between growth stimulation of DAPK1 and mTOR1 signaling, which may ultimately affect autophagy, cell survival, or apoptosis.

## DAPK1 and Apoptosis

The process of programmed cell death, or apoptosis, is defined by the regulated destruction of cellular components either in response to extrinsic (e.g., death receptor ligands) or intrinsic (e.g., deoxyribonucleic acid (DNA) damage) stimuli, resulting in death. Inappropriate apoptosis (either too little or too much) is a cause of many human conditions including autoimmune disorders, neurodegenerative diseases, ischemic damage and many types of cancer (Elmore, 2007). The ability to regulate and modulate the life or death of a cell is recognized for its immense therapeutic potential. The apoptotic pathway consists of a series of highly regulated steps, which include activation, propagation, commitment and execution, are carried out by a diverse array of signal transduction mechanisms including protein kinase activity and subcellular localization via protein association domains (Larner, 2000), such as those found in DAPK1 (Deiss et al., 1995; Cohen et al., 1997).

In a number of cell culture models, DAPK1 has been implicated in pathway leading to apoptosis and in some cases may function prior to the commitment steps of the signal transduction mechanism (Cohen et al., 1999; Raveh et al., 2001; Jang et al., 2002; Pelled et al., 2002; Yamamoto et al., 2002). Thus some of the apoptotic properties of DAPK1 observed in cultured cells will be responsible for certain aspects of human diseases.

A transforming growth factor (TGF)-β is related to DAPK1-regulated pathways and apoptosis, which can stimulate apoptosis in developing neurons and other tissues (De Luca et al., 1996; Schuster and Krieglstein, 2002). Through a cell surface TGF-β receptor, TGF-β initiates cellular actions and transmit the signal inside the cell through Smads (Nakao et al., 1997), signaling molecules that translocate to the nucleus and modulate gene expression (Schuster and Krieglstein, 2002). The upregulation of DAPK1 gene expression occurs due to TGF-β exposure, requiring the function of Smad2, -3 and -4. In Hep3B hepatoma cells, the DAPK1 gene expression was upregulated within 8 h, consistent with the time course of TGF-β-induced apoptosis, which is apparent between 8 and 18 h, suggesting that DAPK1 is an effector of the cell death program. Cells that express DAPK1 death domain, are less sensitive to TGF-β-induced apoptosis, suggesting that DAPK1 substrate phosphorylation mediates TGF-β death signals and its death domain plays a role in facilitating this (Jang et al., 2002). Despite the fact that DAPK1 mediated apoptosis in response to TGF-β is unclear; it is well known that cytochrome c release from mitochondria causing apoptotic protease activating factor 1 (Apaf1) activation and pro-caspase 3 cleavage precedes TGF-β-induced apoptosis (Green and Reed, 1998; Freathy et al., 2000).

DAPK1 has also been reported to mediate TNF-α and IFN-γ induced apoptosis as well as by activation of the Fas receptor (Cohen et al., 1999). Knockdown of DAPK1 by antisense RNA protects HeLa cells from cell death induced by IFN-γ and Fas. In addition, IFN-γ induced apoptosis is decreased by expression of catalytically inactive DAPK1 (Cohen et al., 1997). Cell death induced by p55 TNF-receptor activation is also reduced by the expression of the DAPK1 death domain (Cohen et al., 1999), which supports the role for this domain in mediating cytokine-induced apoptosis. However, cell death is not completely abrogated by either a death domain dominant negative or a catalytically-inactive dominant negative, suggesting that perhaps the death domain helps in localizing DAPK1 within the cell so the catalytic activity of DAPK1 can function in signaling networks (Schumacher et al., 2002).

In addition, many apoptotic signals for neurons, such as excitotoxicity or exposure to DNA-damaging agents, invoke cell death via the p53 pathway (Komarova and Gudkov, 2001). Cell culture models have implicated DAPK1 in cytokine-independent apoptosis via the p53 pathway (Raveh et al., 2001), which has many functions including an active role in neuronal regulation and development (Komarova and Gudkov, 2001), and tumor suppression. Dissociation of p53 from the mouse double minute 2 homolog (Mdm-2) protein and translocation into nucleus cause the activation of p53 (Sherr, 1998). This occurs when alternate reading frame (ARF) protein, human p14^ARF^ or mouse p19^ARF^, interacts with Mdm-2, causing its dissociation from p53 (Sherr, 2001). It has been reported that DAPK1 activates the p53 pathway via p19^ARF^ through an undetermined mechanism. In mouse embryonic fibroblasts (MEFs), overexpression of constitutively active DAPK1 increases levels of p53, p53-regulated proteins Mdm-2 as well as p21, causing apoptosis (Raveh et al., 2001). A DAPK1 construct, containing either a catalytically-inactive mutation of the kinase domain or a deletion of the death domain, are unable to induce cell death in rat embryonic fibroblasts (REFs) as a wild type DAPK1 (Raveh et al., 2001), suggesting that DAPK1 mediates its effects on the p53 apoptotic pathway via its death domain interaction and kinase activity, as it does in the cytokine pathways. In DAPK1 or p53 knockout mice, the reduction of apoptosis suggests that DAPK1 might be functioning upstream of the commitment step in the p53 pathway, shows DAPK1 as a potential target for the modulation of p53-mediated apoptosis (Schumacher et al., 2002).

## DAPK1 and Autophagy

Recently, DAPK1 has been shown to be a critical regulator of autophagy, a catabolic process whereby the cell engulfs cytoplasmic organelles and contents in a double membrane vesicle fuses with the lysosome, upon which its contents are degraded. Autophagy serves as a quality control mechanism in the cytoplasm by degrading long-lived proteins and scavenging damaged organelles and misfolded proteins (Yang and Klionsky, 2010). Autophagic activity is greatly enhanced, under stress conditions (e.g., starvation, oxidative stress, hypoxia,), to facilitate removal of damaged organelles and proteins and/or provide a source of recycled macromolecular building blocks during nutrients deprivation and lack of energy. DAPK1 has been recognized as a mediator of autophagic cell death by inducing a non-apoptotic caspase-independent programmed cell death and promoting the formation of autophagosome via increasing the autophagy flux (Inbal et al., 2002; Zalckvar et al., 2009; Eisenberg-Lerner and Kimchi, 2012).

Expression of DAPK1 leads to the enhanced formation of autophagosomes in various cell types, which was observed as an increase in the appearance of double membrane vesicles enclosing cytoplasmic contents, indicates the varying states of maturation of autophagosomes and autolysosomes (Inbal et al., 2002).

Activation of DAPK1, by various stimuli that induce autophagy, has been shown to involve several inter-related mechanisms that include binding of Ca^2+^-activated CaM to the CaM regulatory domain, (Cohen et al., 1997) dephosphorylation of Ser308 by the PP2A phosphatase within the CaM regulatory domain (Shohat et al., 2001; Gozuacik et al., 2008; Guenebeaud et al., 2010; Widau et al., 2010), and potentially, hydrolysis of GTP to GDP by the ROC-COR domains (Carlessi et al., 2011).

**Figure 3:**
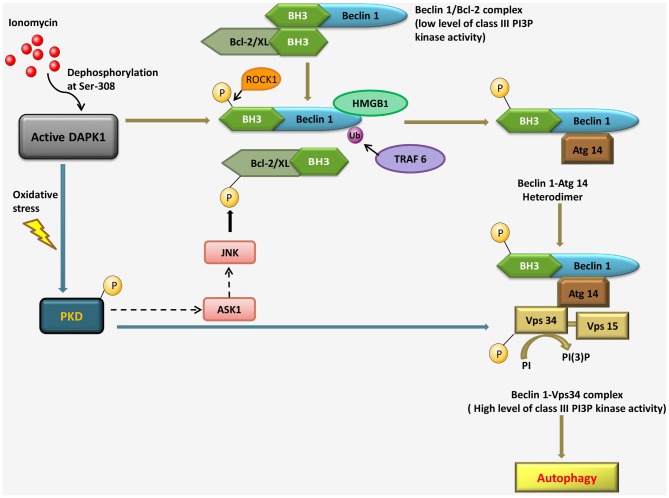
**Pathways linking DAPK1 to the nucleation step of autophagy through the Beclin 1-Vps34 complex formation in response to ionomycin and under oxidative stress**.

Activated DAPK1 positively contributes to the induction stage of autophagosome nucleation via modulating the Vps34 class III phosphatidyl inositol 3-kinase complex by two independent mechanisms. The first, a kinase cascade, involves phosphorylation of protein kinase D (PKD) by DAPK1, which further phosphorylates and activates Vps34. In the second mechanism, DAPK1 directly phosphorylates Beclin1, a necessary component of the Vps34 complex, thereby releasing it from its inhibitors, B-cell lymphoma 2 (Bcl-2). Bcl-2 binds and inhibits Beclin 1, preventing its association with the Vps34 complex. DAPK1 disrupts the Beclin 1/Bcl-2 interaction, in response to ionomycin, via phosphorylation of Beclin 1 within the Bcl-2 homology domain 3 (BH3) domain. In response to starvation, a Ser/Thr rho-associated, coiled-coil-containing protein kinase 1 (ROCK1) can also phosphorylate this residue. DAPK1 phosphorylates PKD, under oxidative stress, which in turn phosphorylates and activates Vps34 directly. Activated Vps34 proceeds to phosphatidyl inositol-3 phosphate (PI3P) formation, which recruits PI3P effector such as Double FYVE-domain containing protein 1 (DFCP1), leading to autophagy. The interaction of Beclin 1 with Bcl-2 interaction can be disrupted by c-Jun N-terminal kinase (JNK)-mediated phosphorylation of Bcl-2, binding of high mobility group box 1 (HMGB1) to Beclin 1 or uniquitination of Beclin 1 by TNF receptor-associated factor 6 (TRAF6). During oxidative stress, PKD can lead to JNK phosphorylation through the mitogen-activated protein kinase 3K (MAPK3K) Apoptosis signal-regulating kinase 1 (ASK1); it is not known if and when DAPK1 can modulate Bcl-2 through a PKD-JNK pathway (Figure 3; Levin-Salomon et al., 2014).

The various scenarios, in which DAPK1 was shown to be necessary for autophagy, have another factor in common: they all involved cell death; but in some scenarios (e.g., ER stress, oxidative stress) other death programs were also evident. As a strong case for autophagic cell death was presented, i.e., death was blocked by inhibition of autophagy and occurred in the absence of another death program.

Although there are many regulators of autophagy but DAPK1 is unique in its potential to impact several different stages within the autophagy flux pathway, including autophagosome induction, trafficking and fusion. Some of these regulatory events are more substantiated and proven than others but it is not known what triggers these regulatory mechanisms, and whether one or more are concurrently activated. Nevertheless, by activating and enhancing multiple steps in the process, DAPK1 has shown the potential for being an autophagy “super-activator” (Levin-Salomon et al., 2014). This may resolve its relation specifically with autophagy associated with cell death. Further research into the mechanisms by which DAPK1 regulates autophagy may reveal not only the function of DAPK1, but also the connection between autophagy and cell death.

## Targeting DAPK1 in Cancer and Neurodegenerative Disease

Recent research and reviews highlight communications between cellular organelles under cancerous and neurodegenerative conditions wherein endoplasmic reticulum stress, inflammation, oxidative stress and autophagy are the signaling pathways involved (Scherz-Shouval and Elazar, 2007; Alirezaei et al., 2011; Doyle et al., 2011; Nixon, 2013; Chaudhari et al., 2014). Both cancer and neurodegenerative disease have been linked with misregulated autophagy, and to loss- and gain-of-function of DAPK1, respectively. Thus it will be interesting to determine DAPK1’s autophagic functions contribute to its role in these pathologic conditions.

### DAPK1 and Cancer

Although the relationship between cancer and autophagy is a complex and often contradictory one as during early stages, autophagy has a tumor suppressive function resulting from its ability to suppress accumulation of p62 and the generation of reactive oxygen species, and limit genomic instability in response to metabolic stress and oxidative stress. Autophagy also serves to prevent inflammation and tumor necrosis (Tian et al., 2003; Degenhardt et al., 2006; Karantza-Wadsworth et al., 2007; Mathew et al., 2007, 2009; White et al., 2010; Lorin et al., 2013). In contrast, in later stages, autophagy is necessary for tumor progression, for example by providing nutrients and energy to the poorly vascularized tumor and rapidly dividing tumor. Furthermore, autophagy contributes to the resistance of tumor cells to radiation and chemotherapy treatment by blocking apoptosis and alleviating cellular stress. Significantly, Beclin 1, an autophagy gene has been shown as tumor suppressor. Beclin 1 has been shown to inhibit the functions of deubiquitinating enzymes that in turn affect stability of p53, a master tumor suppressor (Liu et al., 2011). Similarly, DAPK1’s ability to regulate autophagy at different stages and its frequent epigenetic loss in tumor may be connected. It has many different functions that contribute to its ability to suppress metastasis and tumor growth, which include its ability to upregulate p53 upon oncogene expression (Raveh et al., 2001), to induce apoptosis in response to loss of matrix attachment and cytotoxic cytokines such as TNF-α (Inbal et al., 1997; Wang et al., 2002), to activate pyruvate kinase M’s glycolytic function (Mor et al., 2012), and to arrest cell motility and metastasis (Kuo et al., 2006). Thus, it is unclear that as an autophagy inducer, whether or not DAPK1’s function leads to tumor suppression.

### DAPK1 and Neurodegenerative Disease

DAPK1 has also been associated in the pathogenesis of neuronal disorders such as epilepsy (Mor et al., 2012), Alzheimer’s disease (AD; Li et al., 2006) and ischemic brain injury (Velentza et al., 2003). This can be due to its ability to induce neuronal cell death in response to ceramide, ischemia and glutamate toxicity (Pelled et al., 2002; Tu et al., 2010).

Here we can target DAPK1 to prevent ischemic brain damage by disrupting DAPK1-NR2B interaction promotes neuronal death by potentiating functions of “calcium–activated death-signaling proteins” in the NDC. An interference peptide, Tat-NR2B-CT_1292–1304,_ whose primary sequence includes the membrane transducing domain of the HIV1 Tat protein and the DAPK1-binding domain of NR2B, was developed to prevent DAPK1-mediated NR2B subunit phosphorylation by disrupting DAPK1-NR2B interaction, thereby NR2BR activity potentiation, which attenuates excitotoxic neuronal injuries (Lai et al., 2011). DAPK1 is also associated with late-onset Alzheimer’s disease (LOAD). In terms of a possible role in AD, two single nucleotide polymorphisms (SNPs) i.e., rs4878104 and rs4877365 are identified through the genotyping of chromosome 9 in more than 2000 AD patients, located within human DAPK1, suggested that the genetic variation in DAPK1 modulates susceptibility to LOAD (Li et al., 2006). In experimental animals, elevated DAPK1 activity was detected in brains following ischemic injury and in animal and human brain tissue following seizure-related injury (Henshall et al., 2003, 2004). Bioavailable, small-molecule inhibitors of DAPK1 have been developed and these showed some protection in animal models of acute brain injury (Schumacher et al., 2002; Shamloo et al., 2005) and amyloid-induced hippocampal damage (Craft et al., 2004). Recently it has been found that the hippocampal DAPK1 expression is remarkably increased in the brains of AD patients compared with age-matched normal subjects (Kim et al., 2014). Here a novel role of DAPK1 is identified in the regulation of tau protein (Wu et al., 2011), a MT-associated protein, accumulates in AD potentially as a result of posttranslational modifications, such as hyperphosphorylation and conformational changes (Johnson and Stoothoff, 2004). DAPK1 overexpression increases the tau protein stability and phosphorylation at multiple AD-related sites. In contrast, DAPK1 inhibition by genetic knockout or by overexpression of a DAPK1 kinase-deficient mutant decreases the tau protein stability and suppresses its phosphorylation. In addition, inhibition of DAPK1 kinase activity significantly increases the assembly of MTs and accelerates nerve growth factor-mediated neurite outgrowth. As the DAPK1 is genetically linked to LOAD, it has been suggested that DAPK1 is a novel regulator of tau protein abundance, and upregulation of DAPK1 might contribute to tau-related pathologies in AD. Therefore, DAPK1 might be a novel therapeutic target for the treatment of human AD and other tau-related pathologies (Kim et al., 2014). Further it has been reported that DAPK1 phosphorylates tau protein at Ser262 (*p*S^262^) in cortical neurons of stroke mice and either blocking DAPK1-tau interaction by systematic application of a membrane permeable peptide or genetic deletion of kinase domain (KD) in mice (DAPK1-KD^−/−^) protects spine damage and improves neurological functions against stroke insults. As the interaction of DAPK1-KD has been confirmed with the MT repeat binding domain 1 of tau (R1D) consisting of amino acids IGSTENLK, recently, a membrane-permeable peptide is generated by fusing the peptide IGSTENLK to the transduction domain of the HIV TAT protein, named TAT-R1D. Treatment with TAT-R1D (IGSTENLK) results in effective dissociation of DAPK1-tau complexes and significant decrease in the level of *p*S262 causes apparent alleviation of infraction area as well as the neurological deficits induced by cerebral ischemic stroke (Pei et al., 2015). Thus, blocking DAPK1-tau interaction could be a promising target for developing potential therapy for ischemic stroke.

In addition, MT dynamics can be affected by DAPK1’s activation of the MARK kinases, leading to taupathies (Wu et al., 2011), through phosphorylating microtubule associated proteins (MAPs) such as tau. However, the link between DAPK1’s role in autophagy and neuropathologies is still unclear and remains an important area for future research prospects.

## Author Contributions

PS wrote the manuscript. PT and PR designed the study and contributed in manuscript preparation.

## Conflict of Interest Statement

The authors declare that the research was conducted in the absence of any commercial or financial relationships that could be construed as a potential conflict of interest. The reviewer TD and handling Editor declared their shared affiliation, and the handling Editor states that the process nevertheless met the standards of a fair and objective review.

## Abbreviations

DAPK1: Death-Associated Protein kinase 1
Ser/Thr: serine/threonine
IFN: interferon
Ca^2+^/CaM: Ca^2+^/calmodulin
ROC: Ras of complex proteins
COR: C-terminal of ROC
PDB: Protein Data Bank
HRD: His-Arg-Asp
Hsp90: heat shock protein 90
MIB-1: mindbomb E3 ubiquitin ligase 1
DIP-1: DAPK1 interacting protein-1
CHIP: carboxyl terminus of HSC70-interacting protein
GTP: guanosine triphosphate
GDP: guanosine diphosphate
Pin1: peptidyl prolyl isomerase 1
TNF: tumor necrosis factor
TRADD: TNF receptor type 1-associated death domain protein
FADD: Fas-associated protein with death domain
ERK: extracellular signal regulated
BTB: beta–turn-beta
KLHL20: kelch-like protein20
MARK: MT affinity regulating kinase
PKM2: pyruvate kinase isoform M2
UNC5H2: Unc-5 homolog 2
TSC2: tuberous sclerosis 2
Rheb: Ras homolog enriched in brain
mTOR: mechanistic target of rapamycin
TGF: transforming growth factor
Apaf1: Apoptotic protease activating factor 1
Mdm 2: mouse double minute 2 homolog
MEFs: mouse embryonic fibroblasts
ARF: alternate reading frame
REFs: rat embryonic fibroblasts
Bcl-2: B-cell lymphoma 2
BH3: Bcl-2 homology domain 3
ROCK1: rho-associated, coiled-coil-containing protein kinase 1
PKD: protein kinase D
PI3P: phosphatidyl inositol-3 phosphate
DFCP1: Double FYVE-domain containing protein 1
HMGB1: high mobility group box 1
TRAF6: TNF receptor-associated factor 6
JNK: c-Jun N-terminal kinase
MAPK3K: mitogen-activated protein kinase 3K
ASK1: Apoptosis signal-regulating kinase 1
MARKs: microtubule affinity regulating kinases
MAPs: microtubule associated proteins
NDC: neuronal death signaling complex
NMDA: N-methyl-D-aspartate
AD: Alzheimer’s disease
LOAD: late-onset Alzheimer’s disease
MT: microtubule
KD: kinase domain
R1D: repeat binding domain 1
ANP: Phosphoaminophosphonic Acid-Adenylate Ester.
